# Development of 3D printed electrospun vascular graft loaded with tetramethylpyrazine for reducing thrombosis and restraining aneurysmal dilatation

**DOI:** 10.1093/burnst/tkae008

**Published:** 2024-04-08

**Authors:** Yihong Shen, Yanjun Pan, Fubang Liang, Jiahui Song, Xiao Yu, Jie Cui, Guangfang Cai, Mohamed EL-Newehy, Meera Moydeen Abdulhameed, Hongbing Gu, Binbin Sun, Meng Yin, Xiumei Mo

**Affiliations:** State Key Laboratory for Modification of Chemical Fibers and Polymer Materials, Shanghai Engineering Research Center of Nano-Biomaterials and Regenerative Medicine, College of Biological Science and Medical Engineering, No. 2999 North Renmin Road, Songjiang District, Donghua University, Shanghai 201620, PR China; Department of Cardiothoracic Surgery, Shanghai Children’s Medical Center, School of Medicine, Shanghai Jiao Tong University, No. 1678 Dongfang Road,Pudong New Area, Shanghai 200127, PR China; Department of Cardiothoracic Surgery, Shanghai Children’s Medical Center, School of Medicine, Shanghai Jiao Tong University, No. 1678 Dongfang Road,Pudong New Area, Shanghai 200127, PR China; State Key Laboratory for Modification of Chemical Fibers and Polymer Materials, Shanghai Engineering Research Center of Nano-Biomaterials and Regenerative Medicine, College of Biological Science and Medical Engineering, No. 2999 North Renmin Road, Songjiang District, Donghua University, Shanghai 201620, PR China; State Key Laboratory for Modification of Chemical Fibers and Polymer Materials, Shanghai Engineering Research Center of Nano-Biomaterials and Regenerative Medicine, College of Biological Science and Medical Engineering, No. 2999 North Renmin Road, Songjiang District, Donghua University, Shanghai 201620, PR China; State Key Laboratory for Modification of Chemical Fibers and Polymer Materials, Shanghai Engineering Research Center of Nano-Biomaterials and Regenerative Medicine, College of Biological Science and Medical Engineering, No. 2999 North Renmin Road, Songjiang District, Donghua University, Shanghai 201620, PR China; State Key Laboratory for Modification of Chemical Fibers and Polymer Materials, Shanghai Engineering Research Center of Nano-Biomaterials and Regenerative Medicine, College of Biological Science and Medical Engineering, No. 2999 North Renmin Road, Songjiang District, Donghua University, Shanghai 201620, PR China; Department of Chemistry, College of Science, King Saud University, P.O. Box 2455, Riyadh 11451, Saudi Arabia; Department of Chemistry, College of Science, King Saud University, P.O. Box 2455, Riyadh 11451, Saudi Arabia; Department of Cardiovascular Surgery, Shanghai General Hospital, Shanghai Jiao Tong University School of Medicine, No. 650 Xinsongjiang Road, Songjiang District, Shanghai 201600, PR China; State Key Laboratory for Modification of Chemical Fibers and Polymer Materials, Shanghai Engineering Research Center of Nano-Biomaterials and Regenerative Medicine, College of Biological Science and Medical Engineering, No. 2999 North Renmin Road, Songjiang District, Donghua University, Shanghai 201620, PR China; Department of Cardiothoracic Surgery, Shanghai Children’s Medical Center, School of Medicine, Shanghai Jiao Tong University, No. 1678 Dongfang Road,Pudong New Area, Shanghai 200127, PR China; State Key Laboratory for Modification of Chemical Fibers and Polymer Materials, Shanghai Engineering Research Center of Nano-Biomaterials and Regenerative Medicine, College of Biological Science and Medical Engineering, No. 2999 North Renmin Road, Songjiang District, Donghua University, Shanghai 201620, PR China

**Keywords:** 3D printing, Electrospinning, Tetramethylpyrazine, Thrombosis, Aneurysmal dilatation, Vascular graft

## Abstract

**Background:**

Small-diameter vascular grafts have become the focus of attention in tissue engineering. Thrombosis and aneurysmal dilatation are the two major complications of the loss of vascular access after surgery. Therefore, we focused on fabricating 3D printed electrospun vascular grafts loaded with tetramethylpyrazine (TMP) to overcome these limitations.

**Methods:**

Based on electrospinning and 3D printing, 3D-printed electrospun vascular grafts loaded with TMP were fabricated. The inner layer of the graft was composed of electrospun poly(L-lactic-co-caprolactone) (PLCL) nanofibers and the outer layer consisted of 3D printed polycaprolactone (PCL) microfibers. The characterization and mechanical properties were tested. The blood compatibility and *in vitro* cytocompatibility of the grafts were also evaluated. Additionally, rat abdominal aortas were replaced with these 3D-printed electrospun grafts to evaluate their biosafety.

**Results:**

Mechanical tests demonstrated that the addition of PCL microfibers could improve the mechanical properties. *In vitro* experimental data proved that the introduction of TMP effectively inhibited platelet adhesion. Afterwards, rat abdominal aorta was replaced with 3D-printed electrospun grafts. The 3D-printed electrospun graft loaded with TMP showed good biocompatibility and mechanical strength within 6 months and maintained substantial patency without the occurrence of acute thrombosis. Moreover, no obvious aneurysmal dilatation was observed.

**Conclusions:**

The study demonstrated that 3D-printed electrospun vascular grafts loaded with TMP may have the potential for injured vascular healing.

Highlights

- 3D-printed electrospun vascular grafts loaded with TMP (PCL-PLCL-TMP) were prepared via 3D printing and electrospinning.
- PCL-PLCL-TMP grafts exhibited good hemocompatibility and cytocompatibilty.
- PCL-PLCL-TMP grafts were successfully transplanted into rat abdominal aorta and maintained patency without acute thrombosis formation within 6 months.

## Background

Cardiovascular disease has become a serious human health burden. Statistically, cardiovascular disease causes >18 million deaths annually and has become one of the major causes of morbidity and death worldwide [1,2]. When severe stenosis or occlusion occurs, the diseased vessels need to be replaced, so that coronary artery disease or peripheral vascular disease can be treated. Autologous blood vessels are the gold standard for vascular implantation. However, the limited source of autologous vessels limits their clinical application. Developments in vascular tissue engineering have provided a novel route for the treatment of vascular disease, so it is considered to be one of the best ways to solve the above problems [3,4].

Thrombosis is still an important factor affecting the success of surgery with small-diameter vascular grafts. As an implant directly contacts blood, an artificial vascular graft is regarded as a foreign body, which may lead to activation of the blood coagulation system and inflammation. In clinic, thrombus occlusion in the early stage after implantation is the main cause of vascular graft transplantation failure [5–7]. Therefore, it would be ideal to endow vascular grafts with anticoagulant properties. Ligusticum chuanxiong is a traditional Chinese medicine and is one of the most widely used and oldest herbal medicines in China [8,9]. According to previous study, it has the effect of promoting blood circulation and removing blood stasis in treating heart and blood vessel disease [9]. Tetramethylpyrazine (TMP) is an alkaloid monomer and one of the active ingredients that has been extracted from Ligusticum chuanxiong [10]. Previous research has confirmed that TMP has excellent pharmacological properties for cardiovascular applications, such as antiplatelet aggregation, improved microcirculation and ventricular remodeling inhibition effect [11]. Abnormal platelet activation in blood vessels can cause the gradual release of adenosine diphosphate (ADP) and thromboxane B2 (TxB2), which promote platelet adhesion, activation and aggregation. TMP can effectively treat thrombotic vascular diseases by inhibiting the Phosphatidylinositol 3-kinase (PI3K)/(protein kinase B) AKT signaling pathway, inhibiting platelet aggregation and platelet TxB2 secretion induced by ADP, thus inhibiting abnormal platelet activation [12]. Therefore, these findings suggest that TMP is a promising drug for graft coagulation.

In addition to blood clotting, aneurysmal dilation is another disappointing failure after vascular graft transplantation [13,14]. Although polyester artificial vascular grafts offer a route for blood vessel transplantation, they still encounter difficulty in maintaining a stable tubular structure and resistance to arterial pressure during long-term implantation due to their limited mechanical strength, resulting in adverse events such as aneurysmal dilatation [15–17]. Currently, the development of mechanically reinforced vascular grafts is receiving increasing attention. Zhu *et al*. [18] combined weaving techniques with thermally induced phase separation to develop a mechanically reinforced small-diameter graft. The introduction of a weaved structure can help to maintain structural stability and the graft showed good patency after 6 weeks of implantation. However, these studies were limited by the short duration of implantation. The effectiveness of preventing aneurysmal dilatation in the long-term after implantation should be investigated.

**Figure 1:**
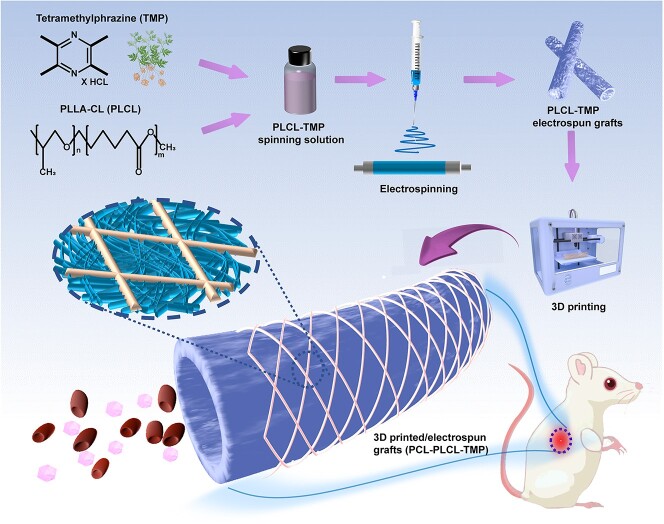
Schematic illustration of 3D printed electrospun vascular graft loaded with TMP. *PCL* polycaprolactone, *PLCL* poly(L-Lactic-co-caprolactone), *TMP* tetramethylpyrazine

The goal of this study was to develop a 3D-printed electrospun mechanically enhanced artificial vascular graft. Due to its excellent biocompatibility, poly(L-lactic-co-caprolactone) (PLCL) has been approved by the FDA for clinical application [19,20]. For vascular tissue engineering, electrospinning has been popular in recent decades for the production of nanoscale fibers. Nanofibrous tubes have high porosity and excellent specific surface area [21]. Hence, electrospinning is a reliable method for constructing artificial vascular grafts. In addition, combining nanofibers and micron fiber skeletons can improve the mechanical properties of electrospun nanofibrous grafts by providing additional mechanical support. Presently, 3D printing technology has attracted significant interest due to its accurate and rapid prototyping ability [22–24]. Through precisely controlled 3D printing, microfibers can be deposited on the outer layer of electrospun grafts. The fiber skeletons can be used to provide extra mechanical support to vascular grafts. In this work, we prepared mechanically reinforced vascular grafts by combining electrospinning and 3D printing technology. The blood compatibility and *in vitro* cytocompatibility of the grafts were evaluated. Then, the graft was transplanted into the abdominal aortas of rats to evaluate its biosafety (Figure 1).

## Methods

### Materials

PLCL with an L-lactic acid/ε-caprolactone ratio of 50 : 50 was purchased from Jinan Daigang Biomaterials Inc. (Jinan, China). Hexafluoroisopropanol (HFIP) was purchased from Shanghai Darui Fine Chemicals (Shanghai, China). Polycaprolactone (PCL) was provided from Sigma-Aldrich Co., Ltd (St. Louis, MO, USA). Tetramethylphrazine was obtained from Shanghai Acmec Biochemical Co., Ltd (Shanghai, China). Cell-Counting-Kit-8 (CCK-8) was purchased by Beyotime Biotech Co., Ltd (Shanghai, China). All culture media and reagents were purchased from Gibco Life Technologies Co. Ltd (Carlsbad, CA, USA).

### Preparation of vascular grafts

#### Fabrication of electrospun nanofibers loaded with TMP

Electrospun nanofibers loaded with TMP were prepared by electrospinning. In brief, 1 g of PLCL and 5, 10 or 20 mg of TMP were dissolved in 10 ml of HFIP and stirred overnight at 25°C to obtain a homogeneous electrospinning solution. During electrospinning, a positive voltage ranging from 12 to 15 kV was applied to the tip to generate nanofibers. The distance between the collector and the Taylor cone was set to 10–15 cm. The flow rate of the solution was 2 ml/h. Additionally, 316L stainless steel rods were used to collect the nanofibers. After fabrication, the nanofibers were dried overnight in a vacuum drying oven to eliminate any residual organic solvents. The nanofibers created with 5, 10 and 20 mg of TMP/g PLCL were identified as PLCL-TMP-5, PLCL-TMP-10 and PLCL-TMP-20, respectively. Pure PLCL nanofibers were produced using the same methodology without the addition of TMP to serve as negative controls.

#### Preparation of PCL-PLCL-TMP vascular grafts with enhanced mechanical properties

PCL (Molecular Weight 80000) was chosen as the raw material for preparing a mechanically enhanced layer via 3D printing. The process involved fabricating PCL microfibers using the 4-axis printing system. This system was comprised of a rapid prototyping manufacturing system (Bio-Architect® SR; Regenovo Biotechnology Co., Ltd, China) in conjunction with a home-made rotary receiver. The PLCL-TMP-10 nanofibers served as the receiver. To initiate the process, PCL was introduced into the extrusion chamber and preheated to 170–180°C for 15 min. Subsequently, the molten PCL was extruded through the nozzle with a point size of 1.0 mm at a temperature of 160–165°C. The extrusion nozzle moved along the center axis of the rotary receiver at a speed of 2.5 mm/s. The receiver rotated continuously at a rate of 15 revolutions per minute (rpm) (n = 15). The printing process involved three reciprocating movements of the nozzle. The grafts with a mechanically enhanced layer were identified as PCL-PLCL-TMP grafts (stented grafts). The electrospun PLCL-TMP-10 grafts (non-stented grafts) served as negative controls.

### Characterization of vascular grafts

The microstructure and morphology of the PLCL, PLCL-TMP-5, PLCL-TMP-10 and PLCL-TMP-20 nanofibers were examined using a scanning electron microscope (PhenomXL, The Netherlands). Before scanning, a thin layer of gold–palladium was sputter-coated on the samples via a sputter coater (SC7620, Quorum Technologies, USA). These samples were characterized via scanning electron microscopy (SEM) at an accelerating voltage of 10 kV. The PCL-PLCL-TMP graft was also observed via SEM. The chemical compositions of PLCL, PLCL-TMP and TMP were analyzed via a Fourier transform infrared spectrometer (ATR-FTIR, Nicolet 6700, USA). Diamond was used as the applied crystal and 32 scans were performed in absorption mode at a resolution of 4 cm^−1^ in the range 4000–400 cm^−1^. To evaluate the release behavior of TMP, different samples were immersed in phosphate-buffered saline (PBS, pH 7.4) at 37°C. At predetermined time points, a sample of the solution was retrieved and an equal volume of fresh medium was added. The concentration of released TMP in each sample was determined via UV–vis spectroscopy at 296 nm. Analysis of the apparent water contact angle of the PLCL, PLCL-TMP-5, PLCL-TMP-10 and PLCL-TMP-20 nanofibers was carried out by using a contact angle analyzer (OCA40, Data-Physical, Germany) three times. For this, 50 μl of distilled water was dropped on the surface of the samples. The test system’s software was subsequently used to capture a photograph of the water droplets on the sample.

### Mechanical properties assays

The mechanical properties of vascular grafts were evaluated with a tensile testing machine (HY-940FS, Shanghai Hengyu Instrument Co., Ltd, China) with a sensor with a loading range of 0–200 N. The axial tensile properties of PLCL, PLCL-TMP-5, PLCL-TMP-10 and PLCL-TMP-20 were examined respectively. Compression performance was measured in accordance with the standard ISO25539-2012. PLCL-TMP grafts (non-stented grafts) and PLCL-TMP grafts with PCL microfibers (stented grafts) were compressed for up to 50% of their deformation for 10 cycles at a compression rate of 5 mm/min in loading–unloading fatigue tests. The compression-cycle curve was recorded and the corresponding compression modulus was calculated. The radial tensile force of the grafts was tested by fixing the grafts in the radial direction with two ‘U’-shaped clamps. The specimens were stretched until breakage. To evaluate the mechanical properties of wet samples, the samples were soaked in PBS in advance after which the relevant experiments were carried out. To evaluate the mechanical properties after degradation *in vitro*, specimens were soaked in PBS and subjected to continuous shaking at a speed of 100 rpm and a temperature of 37°C. The testing samples were retrieved for relevant mechanical tests at the expected point of time (6 months). The three-point bending test was also conducted in accordance with the standard ASTM F2606–08.

### Evaluation of blood compatibility

A 2% (v/v) erythrocyte suspension was used to conduct an *in vitro* hemolysis test. The erythrocyte suspension was obtained from New Zealand rabbits by centrifuging fresh whole blood at 3000 rpm for 10 min. Each testing sample was placed in a sterile container, followed by preheating in 10 ml of saline at 37°C for 30 min. Subsequently, the prepared erythrocyte suspension was dropped into the preheated testing sample and gently mixed. After incubating at 37°C for 1 h, intact erythrocytes were obtained by centrifuging the mixture at 3000 rpm. The absorbance of the supernatant was measured at a wavelength of 540 nm. Deionized water was designated as the positive control and 0.9% normal saline served as a negative control.

To evaluate platelet adhesion, platelet-rich plasma (PRP) supernatant was obtained from whole blood after centrifugation. Then, 500 μl of PRP was incubated with the testing samples at 37°C for 2 h. After incubation, platelets were fixed. Subsequently, the testing samples were rinsed with warm PBS solution to remove nonadherent platelets. The testing samples were then fixed with paraformaldehyde for 2 h and rinsed twice with distilled water. To dehydrate the testing samples, a graded series of ethanol solutions with increasing concentrations (30, 50, 70, 80, 90 and 100%) were used, after which the samples were freeze-dried. Eventually, the morphology of the testing samples was observed via SEM.

The anticoagulant efficacies of the various materials were assessed using prothrombin time (PT) and activated partial thromboplastin time (APTT) assays. Platelet-poor plasma supernatant was obtained from whole blood after centrifugation. The testing groups were incubated in 100 μl of platelet-poor plasma at 37°C for 2 min. To determine the PT, the samples were incubated with PT reagent (100 μl), after which the relevant PT was measured. To determine the APTT, 100 μl of APTT was incubated with the above samples at 37°C, followed by the addition of 0.025 μmol/l CaCl_2_ solution (100 μl). The APTT was then recorded.

### Evaluation of cytocompatibility

To assess cell viability, the samples (14 × 14 mm) were utilized. In our study, the PLCL, PLCL-TMP-5, PLCL-TMP-10 and PLCL-TMP-20 nanofibers were securely placed in 24-well plates using stainless steel rings. Before cell seeding, all samples were subjected to 12 h of UV irradiation following 24 h of treatment with 75 vol% alcohol. The distance between the light source and the samples was set to 20 cm. After sterilization, the samples were rinsed three times with PBS. Subsequently, 300 μl of cell culture medium was added to each well. The testing samples were then incubated at 37°C for 4 h in an atmosphere containing 5% CO_2_. Human umbilical vein endothelial cells (HUVECs) were seeded onto the samples at a density of 2 × 10^4^ cells per well. After seeding, 200 μl of cell culture medium was added. In order to assess the proliferation of HUVECs, Dulbecco's Modified Eagle's Medium containing 10% CCK-8 reagent was replaced with cell culture medium after 1, 4 or 7 days of incubation. Following 2 h of incubation, 100 μl of the solution was transferred to a 96-well plate. A microplate reader (Multiskan MK3, Thermo, USA) was used to measure the absorbance at 450 nm.

Furthermore, to detectg the condition of HUVECs on the samples, the samples were placed in a 24-well culture plate and incubated with 300 μl of cell culture medium at 37°C for 4 h. HUVECs were seeded onto the samples at a density of 2 × 10^4^ cells per well, followed by the addition of a further 200 μl of cell culture medium. After an interval of 4 or 7 days, a live cell staining kit (Beyotime, China) was utilized to stain the living cells according to the manufacturer’s instructions. A fluorescence microscope (DMi8, Leica, Germany) was used to detect living cells. For cell fixation, the samples were incubated with paraformaldehyde for several hours. After rinsing with distilled water, the fixed samples were dehydrated using a series of graded ethanol solutions, and subsequently freeze-dried. Finally, the samples were sputter-coated with gold–palladium and the morphology of HUVECs was observed via SEM.

### *In vivo* assessments

All animal experiments were approved by the Animal Ethics Committee of Shanghai Children’s Medical Center, Shanghai Jiao Tong University School of Medicine. The experiment strictly followed the guidelines for the Care and Use of Laboratory Animals from the National Institutes Health (NIH Publication N01-OD-4-2139, Rev.2).

A total of 9 male Sprague–Dawley rats (weighing ~250–300 g) were used as abdominal aorta replacement models. These rats were divided into three groups: PLCL, PLCL-TMP and PCL-PLCL-TMP. Considering the results of cell proliferation, PLCL-TMP-10 nanofibers were chosen as PLCL-TMP groups. Briefly, Sprague–Dawley rats were anesthetized via inhalation of the anesthetic isoflurane. The animals were breathing spontaneously during surgery. The thick hair around the abdominal region was shaved with depilatory cream. After sterilization and skin preparation, the abdominal aorta was exposed and separated via a median incision. Heparin (300 IU/kg) was utilized for anticoagulation before transplantation. A segment of the abdominal artery (~0.8 cm in length) was then transected between two vascular clamps. The PLCL, PLCL-TMP and PCL-PLCL-TMP grafts (~2.0 mm in inner diameter, ~0.8 cm in length) were implanted respectively and sewn into the defect in the rat abdominal aorta in an end-to-end fashion. After removing the clamps, blood flow was restored and the incision was sutured with 4–0 absorbable sutures.

At predetermined time points, rats were anesthetized according to previous protocols. Doppler sonographic assessment (Affiniti 50, Philips Ultrasound, Inc.) was used to determine the efficiency of the implanted grafts. To evaluate vascular regeneration, rats were sacrificed by injecting an overdose of isoprene barbiturate into the ear margin vein at determined time points. The implanted grafts were harvested and evaluated by hematoxylin–eosin (H&E) staining, Masson trichrome staining, SEM and immunofluorescent staining.

### Statistical analysis

The data are presented as the mean ± standard deviation. The data were analyzed by one-way Analysis of Variance (ANOVA) with Tukey’s *post hoc* test. When comparing two groups, a t-test was used to assess the differences. The CCK-8 results were analyzed by two-way ANOVA with Tukey’s *post hoc* tests. Statistical significance was considered at *p* < 0.05 (* indicates *p* < 0.05).

## Results

### Characterization of the vascular grafts

**Figure 2:**
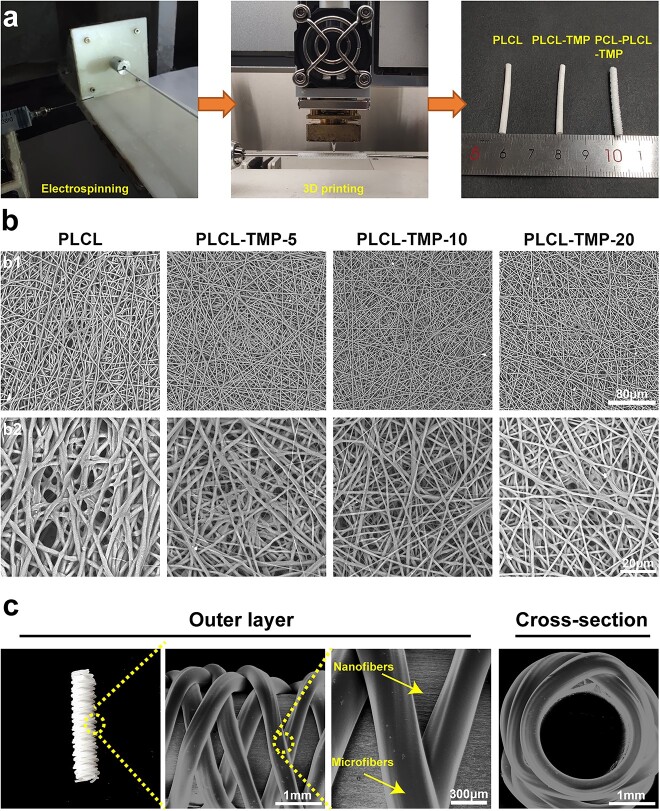
Preparation and structures of 3D- printed electrospun vascular grafts. (**a**) PLCL solution was electrospun and collected on a stainless-steel rod to obtain nanofibrous grafts. Then PCL microfiber was deposited on the nanofibrous grafts via 3D printing. Eventually 3D-printed electrospun vascular grafts were obtained. (**b**) SEM micrographs of electrospun nanofibers (b1) at a magnification of 1000X (scale bar: 80 μm) and (b2) at a magnification of 3000X (scale bar: 20 μm). (**c**) SEM micrographs of 3D printed mechanically reinforced nanofibrous grafts. Scale bars: 1 mm, 300 μm. *SEM* scanning electron microscope, *PCL* polycaprolactone, *PLCL* poly(L-Lactic-co-caprolactone), *TMP* tetramethylpyrazine

We prepared PLCL nanofibers loaded with TMP by electrospinning as the inner layer of vascular grafts. Then the PCL microfibers in the outer layer were further constructed by 3D printing. As shown in Figure 2a, PLCL and TMP could be dissolved in HFIP to form a uniform electrospinning solution, and then a voltage was applied to collect nanofibers on a rotating stainless-steel rod to obtain nanofibrous tubular materials. Afterwards, through the reciprocating movement of the printer, the PCL microfibers were deposited on the nanofibrous tubular materials. The basic parameters of the samples are shown in Table S1, see online supplementary material. The morphologies of PLCL and PLCL-TMP nanofibers are shown in Figure 2b. The luminal surfaces of the vascular grafts were composed of disordered, smooth and uniform nanofibers, with structures similar to that of the natural extracellular matrix. After the addition of TMP, the inner vascular layer maintained a good fibrous structure. As can be seen from the SEM images of the outer surface of PCL-PLCL-TMP grafts (Figure 2c), well-organized PCL fibers were attached to the surface of the nanofibers. The stable interlaced structure maintained the outer tubular structure.

**Figure 3:**
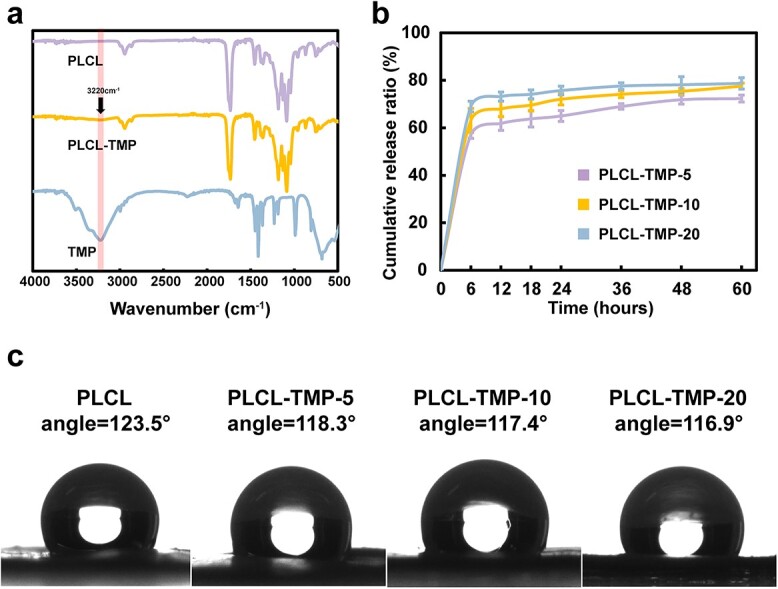
Characterization of samples. (**a**) The FTIR spectra of PLCL, PLCL-TMP and TMP. (**b**) Release profile of TMP from PLCL-TMP nanofibers. (**c**) Water contact angles of the samples. *FTIR* fourier transform infrared spectroscopy, *PCL* polycaprolactone, *PLCL* poly(L-lactic-co-caprolactone), *TMP* tetramethylpyrazine

As shown in Figure 3a, the characteristic absorption peaks of the PLCL nanofibers were at 1756 and 1735 cm^−1^. The characteristic peak at 3220 cm^−1^ appeared in the PLCL-TMP group, which indicated that TMP had been added to the PLCL nanofibers. Additionally, the TMP release ratio is shown in Figure 3b. The curves of drug release from PLCL scaffolds containing 5, 10 and 20% TMP (PLCL-TMP-5, PLCL-TMP-10 and PLCL-TMP-20, respectively) exhibited tiny standard deviations, which confirmed the uniform distribution of TMP within them. During the first 6 h, the release of TMP from scaffolds was 56.98, 63.11 and 68.95% for PLCL-TMP-5, PLCL-TMP-10 and PLCL-TMP-20 groups, respectively. In the following 54 h, all the groups exhibited relatively sustained-release behavior, reaching the release of 72.35, 77.56 and 78.72%, respectively.

As shown in Figure 3c, the hydrophilicities of PLCL, PLCL-TMP-5, PLCL-TMP-10 and PLCL-TMP-20 were determined by contact angle tests. The mean contact angle of each sample was in the range 116.9–123.5° and the materials were hydrophobic. The loading of TMP did not change the hydrophilicity of the vascular grafts.

### Mechanical properties

**Figure 4:**
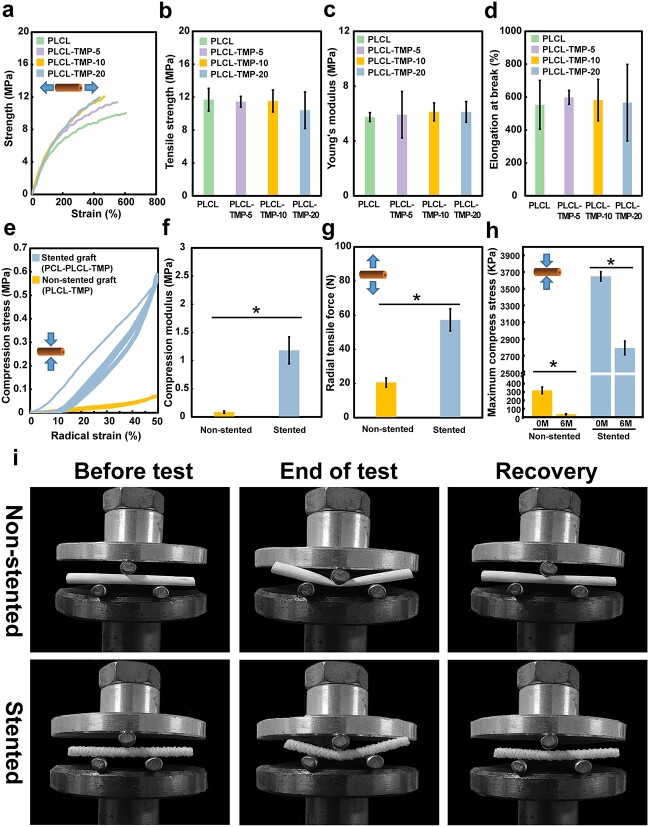
*In vitro* mechanical properties (dry samples). (**a**) Stress–strain curves, (**b**) tensile strength, (**c**) compression modulus and (**d**) elongation at break of the electrospun vascular grafts during axial stretching. (**e**) Typical compression load–displacement curves of samples when they were cyclically compressed to half the initial diameters 10 times. (**f**) Compression modulus of non-stented and stented grafts when radially compressed. (**g**) Radical tensile force during radial stretching. (**h**) Maximum compression stress during 6 months of degradation. (**i**) Images of three-point bending tests. (^*^p < 0.05.) *PCL* polycaprolactone, *PLCL* poly(L-lactic-co-caprolactone), *TMP* tetramethylpyrazine

Good mechanical properties are essential for vascular patency and aneurysm prevention. Insufficient mechanical support may lead to adverse events. The electrospun grafts were flattened into a rectangle shape for axial tensile test. Figure 4a showed the representative stress–strain curve of the nanofibers. The elongation at break and Young’s modulus of the grafts, respectively, were calculated according to the stress–strain curve. As shown in Figure 4b, the tensile stresses at maximum stress were 11.68 ± 1.37, 11.44 ± 0.66, 11.53 ± 1.34 and 10.40 ± 2.22 MPa for PLCL, PLCL-TMP-5, PLCL-TMP-10 and PLCL-TMP-20 groups, respectively. The elongations at break were 553.42 ± 148.06, 598.45 ± 42.30, 581.79 ± 126.10 and 566.28 ± 232.52%, respectively (Figure 4d). The tensile modulus and elongation at break of the nanofibers did not apparently change after drug loading. According to previous studies, the tensile strengths of natural coronary arteries range from ~1.4–11.14 MPa, with the elongation at break usually >40% [25]. The tensile strength of the grafts was comparable to that of the native arteries, while the elongation at break of the samples was much greater than that of natural coronary arteries. Compared with carotid artery (0.83 MPa) [18], the modulus of the PLCL (5.74 ± 0.32 MPa), PLCL-TMP-5 (5.91 ± 1.70 MPa), PLCL-TMP-10 (6.12 ± 0.65 MPa) and PLCL-TMP-20 (6.12 ± 0.75 MPa) was significantly better (Figure 4c).

In addition to the axial mechanical properties, vascular grafts should be able to withstand the pressure and have good radial strength against aneurysm dilatation and rupture. As shown in Figure 4e, the results of the cyclic compression tests proved that the mechanical properties of the nanofibers can be effectively enhanced by the PCL microfibers covering the nanofibers. Under cyclic compression, the stented grafts showed good structural stability. Figure 4f reveals the results of the compression modulus. The modulus of the non-stented grafts was 0.09 ± 0.02 MPa. The modulus of the stented graft was 1.18 ± 0.24 MPa. In addition, the radial tensile forces of the grafts are shown in Figure 4g. The force of the non-stented grafts was 20.57 ± 2.64 N and the force of stented grafts was 57.26 ± 6.45 N. Figure 4h shows the maximum compression stress of stented and non-stented grafts after 6 months of degradation. The degradation process decreased the maximum compression stress in both the stented and non-stented groups. The strength of the stented grafts was much greater than that of the non-stented grafts, indicating that the PCL microfibers may play a mechanical supporting role for long-term transplantation.

The mechanical properties at wet state of vascular grafts are also critical for simulating an *in vivo* experiment. Figure S1, see online supplementary material, shows the *in vitro* mechanical properties (wet samples). As shown in Figure S1a–c, the tensile strength, modulus and elongation at break of the nanofibers will not apparently change after drug loading. Moreover, the mechanical properties (compression force at 50% strain and radial tensile force) of nanofibers could be effectively enhanced by the PCL microfibers covering the nanofibers (Figure S1d–f).

To analyze the bending resistance of the vascular grafts, the three-point bending test was conducted. In Figure 4i, it can be seen that the non-stented grafts had poor bending resistance. After external pressure, a kink appeared in the non-stented graft group, which were unable to maintain their original shape. In contrast, the stented-grafts were bendable and could return to their initial shape at the end-point of the experiment, without obvious injury or permanent deformation. Stented grafts have excellent anti-kink performance, which is a key factor in preventing the occurrence of vortex blood flow and stenosis due to kinking and twisting. In brief, these results proved that the stented grafts have good mechanical properties and can provide structural support for nanofiber artificial vascular grafts to withstand blood pressure.

### Blood compatibility

Considering that vascular grafts are in direct contact with blood, blood compatibility is a key index in cardiovascular devices. Potential issues such as coagulation, hemolysis and thrombosis will affect the success rate of vascular transplantation [26].

**Figure 5:**
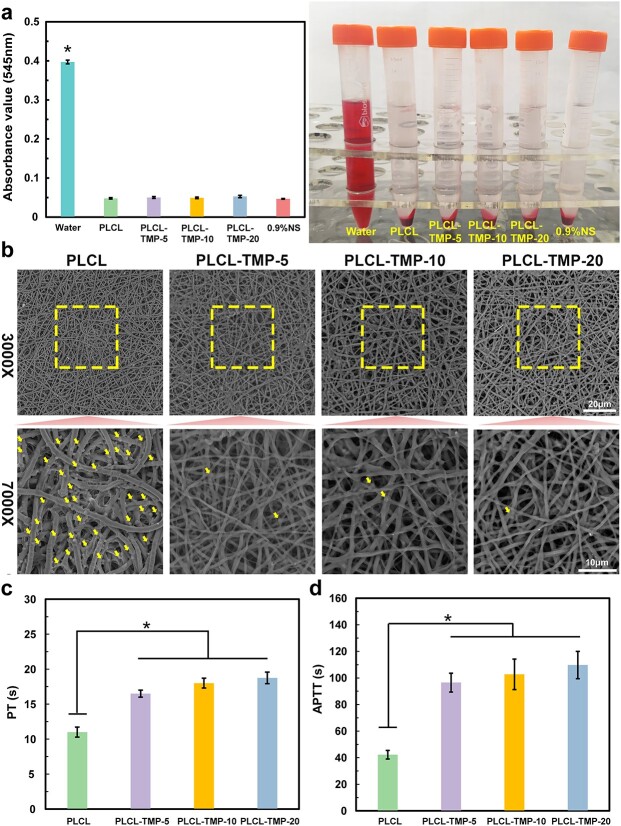
Blood compatibility of grafts. (**a**) Quantification and digital photos of relative hemolysis rate. (**b**) Platelet deposition determined by SEM images; yellow arrows indicates platelets. Scale bars: 20 μm, 10 μm. Coagulation time of PT (**c**) and APTT (**d**). (^*^p < 0.05.) *PT* prothrombin time, *APTT* activated partial thromboplastin time, *PCL* polycaprolactone, *PLCL* poly(L-lactic-co-caprolactone), *TMP* tetramethylpyrazine

The hemolysis test is widely used to evaluate the extent of damage to red blood cells caused by blood contact materials. As shown in Figure 5a, the hemolysis rates of all the grafts were <5%, indicating that the introduction of TMP did not affect the hemolysis characteristics of the grafts. In comparison to PLCL group, it is less likely to cause red blood cell rupture. In accordance with ISO10993-4, these grafts were considered to be nonhemolytic.

When an artificial vascular graft comes into contact with the blood, fibrin is likely to adhere to the lumen area and become deformed. Then, platelets and coagulation systems are easily activated, resulting in lumen stenosis and vascular occlusion [27]. Therefore, the anti-platelet adhesion of grafts is a key property of vascular grafts. A typical SEM image of platelet adhesion on different groups of nanofibers after 2 h of incubation with PRP is shown in Figure 5b. We observed that fewer platelets adhered to the PLCL-TMP nanofibers than to the PLCL nanofibers. This difference may be due to the release of TMP from the nanofibers, thus inhibiting the adhesion of platelets to their surface.

In order to evaluate the anticoagulation effects in the internal and external coagulation pathways, PT and APTT tests were performed. The PT value in the PLCL group (11.0 ± 0.7 s) was significantly lower than that in the PLCL-TMP-5 (16.5 ± 0.5 s), PLCL-TMP-10 (18.0 ± 0.7 s) and PLCL-TMP-20 (18.75 ± 0.8 s) groups (Figure 5c). Similarly, the APTT value in the PLCL group (42.25 ± 3.3 s) was significantly lower than that in the PLCL-TMP-5 (96.5 ± 7.1 s), PLCL-TMP-10 (102.8 ± 11.5 s) and PLCL-TMP-20 (109.8 ± 10.3 s) groups (Figure 5d). These results confirmed that the introduction of TMP could effectively promote the anticoagulant effect of these vascular grafts.

### Cytocompatibility assays

**Figure 6:**
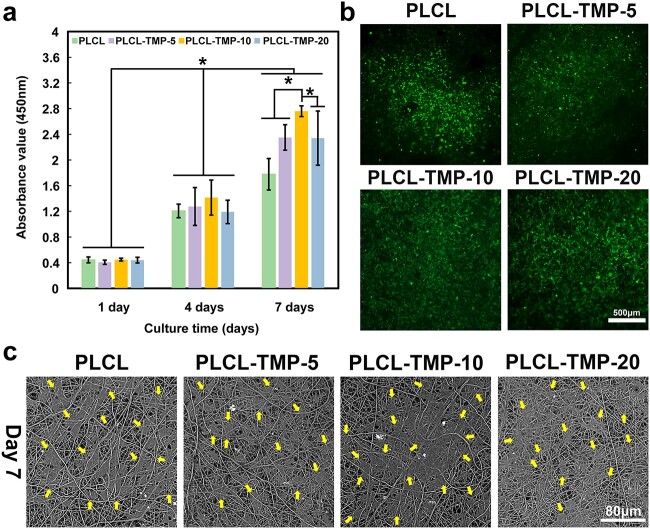
Cytocompatibilty of grafts. (**a**) Proliferation of HUVECs on the surface of nanofibers. (**b**) Fluorescence microscopic images of HUVECs grown on the surface of samples after 7 days (the living cells were stained green). Scale bar: 500 μm (**c**) SEM images of HUVECs cultured on samples after 7 days; yellow arrows indicate cells. Scale bar: 80 μm (^*^p < 0.05.) *HUVECs* human umbilical vein endothelial cells, *PCL* polycaprolactone, *PLCL* poly(L-lactic-co-caprolactone), *TMP* tetramethylpyrazine

The formation of healthy endothelium plays a key role in maintaining the long-term patency of grafts. Within 7 days, HUVECs were cultured on vascular grafts to assess the potential of vascular endothelialization. As shown in Figure 6a, the proliferation of HUVECs on PLCL, PLCL-TMP-5, PLCL-TMP-10 and PLCL-TMP-20 nanofibers was evaluated. The absorbance of CCK-8 in each group increased steadily during the 7 days of cell culture, indicating that all the testing samples supported the proliferation of HUVECs. The CCK-8 results indicated that the PLCL-TMP-10 group had good cytocompatibility. In terms of cell adhesion, calcein-AM staining was used to observe the state of the HUVECs on the samples (Figure 6b). The results showed good cell viability, thereby indicating the good cytocompatibility of the nanofibers. In addition, the morphology of the HUVECs was observed by SEM (Figure 6c). After 7 days of culture, HUVECs were connected into patches, which is mainly attributed to the rapid proliferation of cells.

### Patency

**Figure 7:**
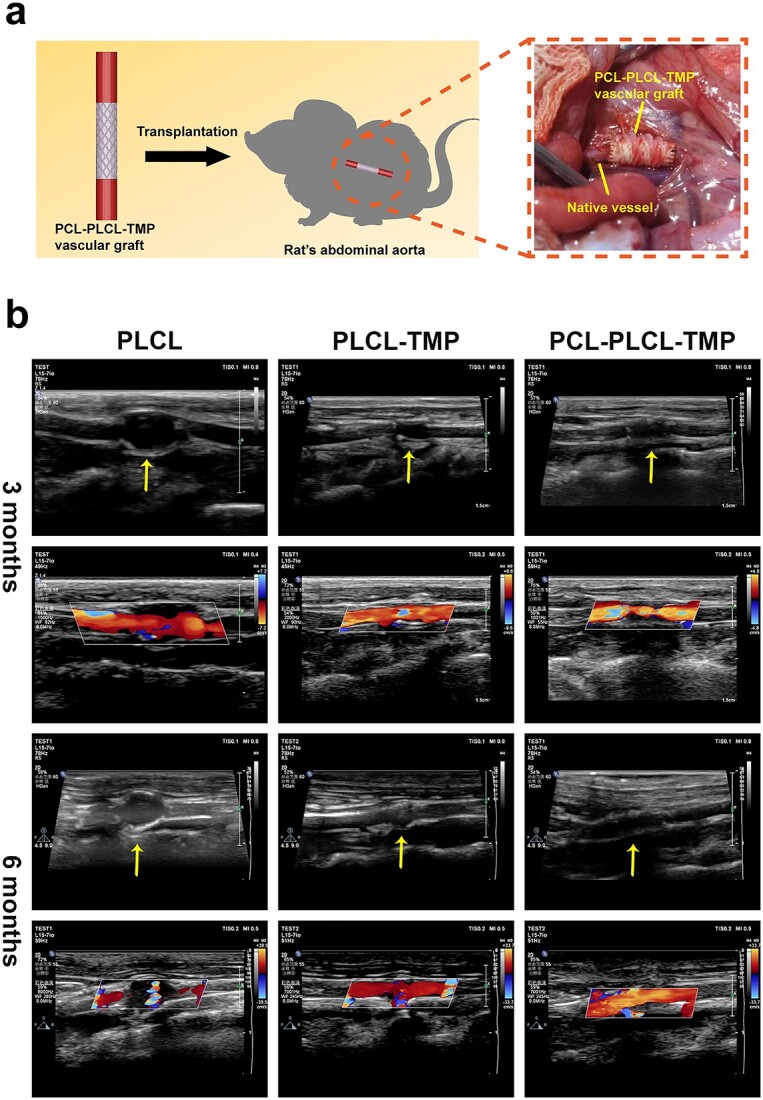
Effect of PLCL, PLCL-TMP and PCL-PLCL-TMP grafts on transplantation in rat abdominal aortas. (**a**) Schematic diagram of PCL-PLCL-TMP vascular grafts implanted into rats’ abdominal aorta. (**b**) Color Doppler ultrasound images of PLCL, PLCL-TMP and PCL-PLCL-TMP grafts at 3 and 6 months after transplantation; yellow arrows indicate the transplantation site. *PCL* Polycaprolactone, *PLCL* poly(L-lactic-co-caprolactone), *TMP* tetramethylpyrazine

For small-caliber artificial vascular grafts, long-term patency is considered to be one of the most important factors affecting the success rate of surgery. The PLCL, PLCL-TMP and PCL-PLCL-TMP grafts were used to replace the abdominal aorta of rats (Figure 7a and S2, see online supplementary material). Considering the results of cell proliferation, PLCL-TMP-10 nanofibers were chosen as the PLCL-TMP groups. Proximal and distal anastomosis after graft transplantation were observed. After replacement, no bleeding was found in the vascular anastomosis in both the experimental group and the control group. Six months after transplantation, the patency of the graft was evaluated by high-resolution ultrasound. Color Doppler ultrasonography showed that all grafts could maintain patency 3 months after transplantation (Figure 7b). Six months after transplantation, vascular lumen dilatation occurred in the PLCL and PLCL-TMP grafts, especially in the PLCL grafts, similar to aneurysms. In the PCL-PLCL-TMP graft, there was no obvious dilation of the vascular lumen and the blood flow signal was clear, indicating that the grafts could withstand the blood pressure of the rat abdominal aorta.

**Figure 8:**
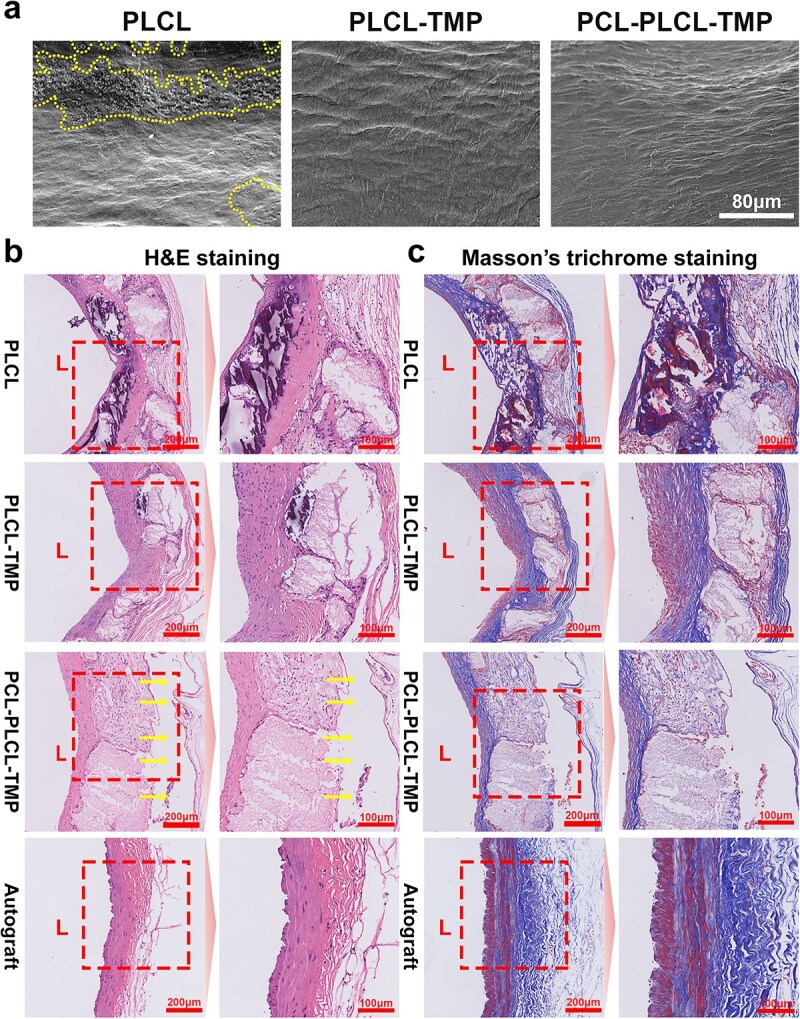
SEM micrographs and histological analysis of grafts. (**a**) SEM micrographs of the lumen surface of the retrieved PLCL, PLCL-TMP and PCL-PLCL-TMP grafts 6 months after transplantation; yellow dashed area indicates the platelets. Scale bar: 80 μm. (**b**) Representative H&E images of PLCL, PLCL-TMP, PCL-PLCL-TMP grafts 6 months after transplantation. Scale bars: 200 μm, 100 μm. (**c**) Masson’s trichrome staining micrographs of PLCL, PLCL-TMP, PCL-PLCL-TMP grafts 6 months after transplantation. Yellow arrows indicate remaining 3D-printed PCL microfibers. Vessel lumen is marked as ‘L’. *H&E* hematoxylin & eosin, *PCL* polycaprolactone, *PLCL* poly(L-lactic-co-caprolactone), *TMP* tetramethylpyrazine

The PLCL, PLCL-TMP and PCL-PLCL-TMP grafts were retrieved 6 months after transplantation for overall evaluation. Endothelial regeneration and platelet adhesion on the inner wall of the graft were observed via SEM (Figure 8a). No obvious platelet adhesion or thrombus matrix was found in the lumen of the PLCL-TMP or PCL-PLCL-TMP grafts. In contrast, a significant number of platelets was found in the lumen of the PLCL grafts. The introduction of TMP may aid in preventing platelet deposition in vascular grafts. Six months after transplantation, the lumen surface of the PCL-PLCL-TMP graft revealed endothelial cell coverage.

### Histological analysis

In order to evaluate the effect of vascular tissue regeneration, we performed histological analysis of intermediate cross-sections via H&E staining (Figure 8b) and Masson’s trichrome staining (Figure 8c). The PCL-PLCL-TMP grafts did not cause stenosis or thrombosis within 6 months, and they maintained their early shape and did not collapse. Six months after transplantation, we observed significant degradation of the PLCL nanofibers, where the fragments of the scaffold were covered by fibrous tissues and cells. Due to the degradation of the material, the graft portion became loose and a large number of voids appeared. The luminal surface of the grafts performed well and the new tissue was regularly arranged.

Collagen provides mechanical support for blood vessels. For the detection of collagen, Masson’s trichrome staining was conducted. In the PCL-PLCL-TMP grafts, large amounts of collagen fibers were observed in the graft lumen. The collagen fibers in the graft lumen were regularly and tightly arranged, indicating the formation of new tissue. The regenerated tissue enclosed the scaffold fragments.

### Immunofluorescence staining

**Figure 9:**
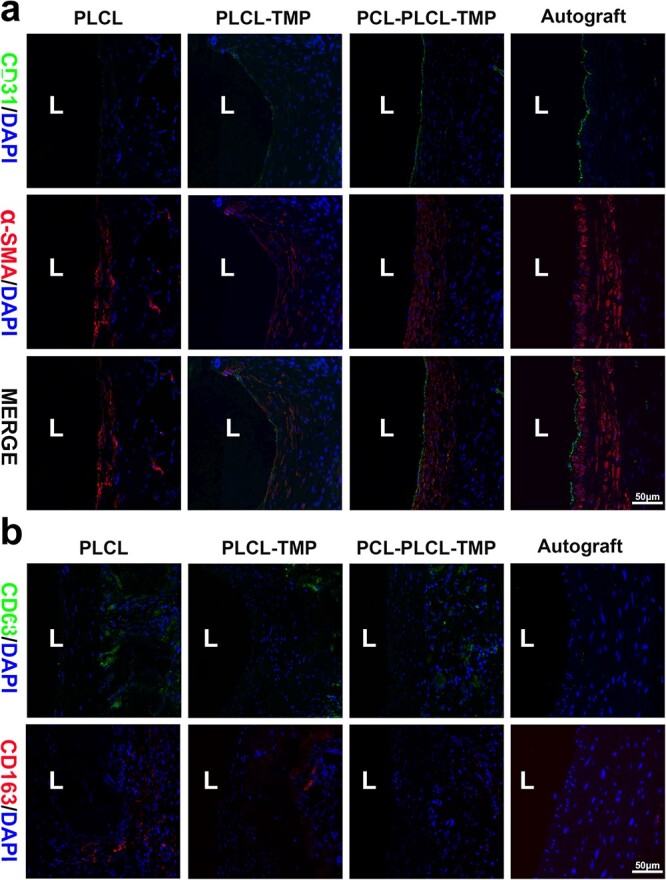
Immunofluorescence staining of the vascular graft. (**a**) Immunofluorescence staining of the vascular graft for CD31 (green), α-SMA (red) and DAPI (blue) 6 months after transplantation. (**b**) Immunofluorescence staining of the vascular graft for CD68 (green) and CD163 (red) 6 months after transplantation. Scale bar: 50 μm. Vessel lumen is marked as ‘L’. *CD31* platelet endothelial cell adhesion molecule-1, *α-SMA* α-smosoth muscle actin, *CD68* cluster of differentiation 68, *CD163* cluster of differentiation 163, *PCL* polycaprolactone, *PLCL* poly(L-lactic-co-caprolactone), *TMP* tetramethylpyrazine

During vascular remodeling, the interactions between endothelial cells and smooth muscle cells (SMCs) play significant roles in promoting the recovery of vascular function. The marker platelet endothelial cell adhesion molecule-1 (CD31) is commonly used to detect the formation of endothelial cell layers, and vascular smooth muscle cells are usually detected by the marker α-smooth muscle actin (α-SMA). As shown in Figure 9a, a high density of endothelial cells was found in the PCL-PLCL-TMP grafts. There was continuous endothelial cell formation on the luminal surface of the regenerated tissue in the PCL-PLCL-TMP grafts, which was similar to that of the native vessels. In addition, α-SMA is often used to detect the formation of SMCs and myofibroblasts. The outer surface of the PCL-PLCL-TMP grafts displayed an evenly distributed layer of SMCs. In terms of the shape, arrangement and density of the regenerated tissue, the SMCs layer of the PCL-PLCL-TMP grafts was similar to that of the native vessels. To evaluate the inflammatory response, immunohistochemical staining for cluster of differentiation 68 (CD68) and CD163 was performed (Figure 9b). Persistent inflammatory injury plays a very important part in stenosis. In comparison to rat abdominal aorta, the inflammatory response was most severe in the PLCL groups, whereas the expression of CD68 and CD163 was decreased in the PLCL-TMP and PCL-PLCL-TMP groups. These results indicate that the introduction of TMP could effectively reduce the persistent inflammatory response on the grafts.

## Discussion

In the clinic, autologous vessels are the gold standard for vascular transplantation. However, a shortage of donors limits their clinical application. Therefore, the development of small-diameter tissue-engineered vascular grafts has important clinical application value and prospects [28,29]. Recently, the design of the grafts has been developed from monolayer structures to multilayer structures with excellent mechanical properties and outstanding anticoagulant properties.

Severe vascular stenosis or occlusion is one of the major reasons for the failure of artificial vascular graft transplantation [30]. Therefore, it is beneficial to use some drugs or proteins to functionalize the artificial vascular grafts. Ligusticum chuanxiong Hort is a traditional Chinese medicine that has been widely used for thousands of years [31]. The introduction of TMP endows grafts with excellent anticoagulant properties, which is beneficial for maintaining the patency of vascular grafts. In our study, we found that PLCL nanofibers loaded with TMP could effectively reduce platelet adhesion *in vitro* (Figure 5b). In terms of this issue, TMP can inhibit the secretion of TxB2 and ADP-induced platelet aggregation by inhibiting the PI3K/AKT signaling pathway [12]. TMP can prevent the abnormal activation of platelets and effectively treat thrombotic vascular diseases. As shown in Figure 8a, the PLCL-TMP and PCL-PLCL-TMP grafts showed less platelet adhesion *in vivo* than the PLCL grafts. Moreover, a previous study demonstrated that the interaction between inflammatory cells and platelets could mediate thrombo-inflammation [32,33]. It was reported that TMP could increase the level of MCPIP1 (an endogenous inflammatory regulatory factor), and decrease the expression of the proinflammatory chemokine monocyte chemoattractant protein-1 and inflammatory factors such as interleukin-6, tumor necrosis factor-α and interleukin-1β, thereby reducing the inflammatory response. We found that the expression of CD68 and CD163 in the PLCL grafts was higher than that of PLCL-TMP and PCL-PLCL-TMP grafts (Figure 9b). The introduction of TMP not only inhibited platelet activation, but also reduced the inflammatory response.

Artificial vascular grafts should have ideal mechanical properties to provide sufficient mechanical support for vascular regeneration. However, the defects in structural design and a lack of good mechanical properties may limit the vascular regeneration effect [34,35]. Blood pressure can cause hyperextension of the vessel wall and periodic stretching of the artery wall, resulting in increased stress in the vessel wall. Insufficient mechanical strength may hinder maintenance of tubular structure and resistance to arterial pressure, which may eventually lead to adverse events such as aneurysmal dilation. In addition, the distortion caused by body movements, intracavitary pressure and external pressure will lead to the kinking of artificial blood vessels, which will lead to blood turbulence and eventually form vascular stenosis [34]. Therefore, imparting grafts with sufficient mechanical properties and kinking resistance is of great significance in the design of grafts. In our study, 3D-printed PCL microfibers served as the mechanical enhancement layer of the PLCL electrospun grafts, which played a mechanical supporting role after transplantation. Compared to traditional electrospun grafts, the introduction of PCL microfiber provided structural mechanical support and bending resistance. The regulatory effects of the 3D-printed PCL microfiber on the mechanical properties of the reinforced grafts can be ascribed to the fusion between fibers [36]. The contact fusion between the fibers increased the mechanical strength of electrospun vascular grafts. The intersection of the PCL fibers was bonded together, which provided mechanical support for the PLCL-based grafts. According to the *in vivo* results, color Doppler ultrasound proved that there was no distinct dilatation of the vascular lumen in the PCL-PLCL-TMP grafts. The blood flow signal was clear and no obvious lumen stenosis was found. In contrast, aneurysmal dilatation was observed in the PLCL and PLCL-TMP grafts (Figure 7b). The PCL fibers maintained the structure of electrospun grafts under the impact of blood flow [37]. In terms of degradation, a previous study showed that PLCL-based grafts were highly degraded after being implanted *in vivo* for 8 months [20]. In contrast, PCL demonstrates a slow degradation time *in vivo* [38]. The slow degradation rate of PCL may help to prevent postoperative adverse events caused by a rapid loss in mechanical properties in the early transplantation stage.

Although our results showed that these PCL-PLCL-TMP grafts could maintain patency and no aneurysmal dilatation 6 months after transplantation in rat abdominal aorta, the current research has some limitations. Firstly, our research was limited to small animals such as rats. The long-term results of PCL-PLCL-TMP grafts in larger, more clinically relevant animal models still need to be further evaluated. In addition, the biomolecular mechanism of 3D-printed structures and TMP in vascular regeneration still needs to be further evaluated. Therefore, our future research will focus on graft transplantation into large animals and analysis of its molecular biological mechanisms.

## Conclusions

Based on electrospinning and 3D printing, 3D-printed electrospun vascular grafts loaded with TMP were successfully fabricated and transplanted into the abdominal aortas of rats to evaluate their biosafety and efficacy. The results proved that the stented graft had excellent flexibility and strong compressive properties. Specifically, the introduction of TMP effectively reduced the adhesion of platelets. The biosafety after transplanting into the abdominal aortas of the rats was evaluated. The PCL-PLCL-TMP grafts showed good biocompatibility and mechanical strength within 6 months and maintained substantial patency without the occurrence of acute thrombosis. These results confirm the potential of 3D-printed electrospun vascular grafts loaded with TMP for clinical application.

## Abbreviations

ADP: Adenosine diphosphate; APTT: Activated partial thromboplastin time; CD31: CD68: Cluster of differentiation 68; CD163: Cluster of differentiation 163; FTIR: Fourier transform infrared spectroscopy; H&E: Hematoxylin–eosin; HFIP: Hexafluoroisopropanol; HUVECs: Human umbilical vein endothelial cells; PBS: Phosphate-buffered saline; PCL: Polycaprolactone; PLCL: Poly(L-lactic-co-caprolactone); PRP: Platelet-rich plasma; PT: Prothrombin time; rpm: Revolutions per minute; SD: Sprague–Dawley; SEM: Scanning electron microscope; α-SMA: α-Smooth muscle actin, TMP: Tetramethylpyrazine; TxB2: Thromboxane B2.

## Funding

This research was supported by the Science and Technology Commission of Shanghai Municipality, China (Nos. 20S31900900, 20DZ2254900) and the Sino German Science Foundation Research Exchange Center, China (M-0263), China Education Association for International Exchange (2022181). It was also supported by the General Project of SHDC (SHDC22021213), Fundamental Research Funds for the Central Universities (No. 2232023D-10). This project was also supported by Researchers Supporting Project Number (RSP2024R65), King Saud University, Riyadh, Saudi Arabia.

## Authors’ contributions

YS: conceptualization, data curation and writing—original draft; YP: methodology and data processing; FL and GC: data curation; JS: methodology; XY: data processing; JC: methodology; ME and MMA: writing, reviewing and editing; HG: supervision; BS and XM: supervision, writing, reviewing and editing, and funding acquisition; MY: supervision and funding acquisition.

## Conflict of interest

None declared.

## Data availability

The authors declare that the data supporting the findings of this study are available within the paper and its supplementary information files.

## Supplementary Material

Figure_S1_S2_and_Table_S1_supporting_information_tkae008
