# Supplementary material for: Development of 3D printed electrospun vascular graft loaded with tetramethylpyrazine for reducing thrombosis and restraining aneurysmal dilatation
Source: Burns Trauma. 2024 Apr 8;12:tkae008. doi: 10.1093/burnst/tkae008 (PMC11002459; doi:10.1093/burnst/tkae008)
Supplement: Figure_S1_S2_and_Table_S1_supporting_information_tkae008 [file figure_s1_s2_and_table_s1_supporting_information_tkae008.docx]

**Support Information**


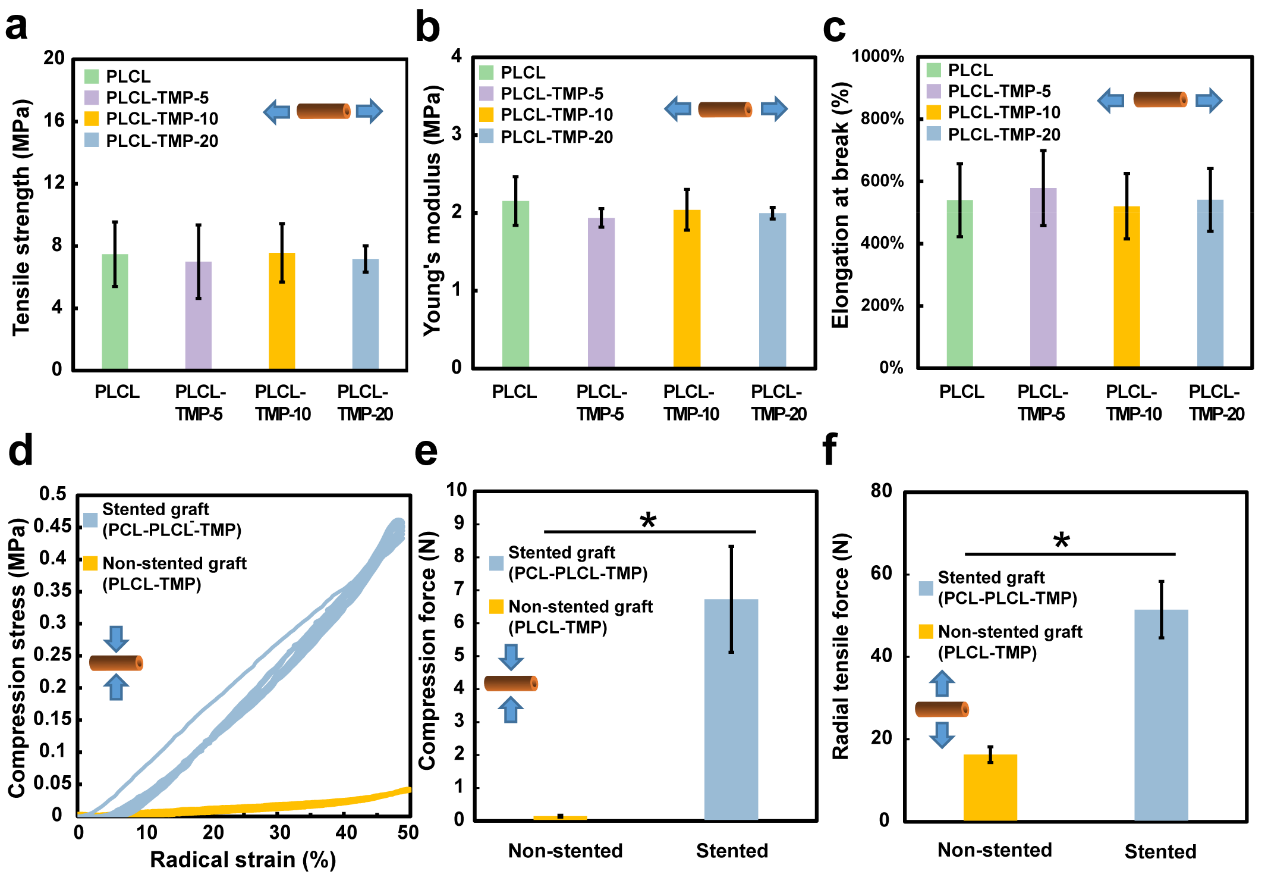


**Figure S1.** *In vitro* mechanical properties (wet samples). (**a**) Tensile strength, (**b**) compression modulus and (**c**) elongation at break of the electrospun vascular grafts during axial stretching. (**d**) Typical compression load-displacement curves of samples when they were cyclically compressed to half the initial diameters for ten times. (**e**) Compression force of non-stented and stented grafts when radially compressed at 50% strain. (**g**) Radical tensile force during radial stretching. (∗*p* < 0.05.) *PCL* polycaprolactone, *PLCL* poly(L-Lactic-co-caprolactone), *TMP* tetramethylpyrazine


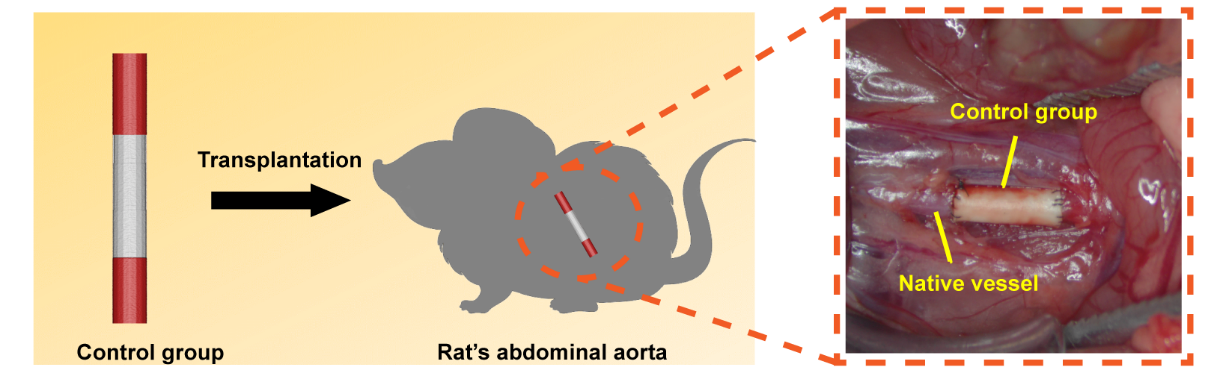


**Figure S2.** Schematic diagram of control groups (PLCL and PLCL-TMP grafts) implanted into rats' abdominal aorta. *PLCL* poly(L-Lactic-co-caprolactone), *TMP* tetramethylpyrazine

**Table S1 Basic parameters of samples**

|  | **Wall thickness**  **(mm)** | **Outer diameter (mm)** | **Pore diameter (μm)** | **Microfiber thickness (μm)** |
| --- | --- | --- | --- | --- |
| **PLCL** | 0.20 ± 0.05 | 2.21 ± 0.05 | 9.12 ± 1.05 | **/** |
| **PLCL-TMP** | 0.20 ± 0.05 | 2.19 ± 0.05 | 9.44 ± 1.44 | **/** |
| **PCL-PLCL-TMP** | 0.52 ± 0.10 | 2.49 ± 0.10 | 9.25 ± 1.87 | 468.3± 56.7 |

*PCL* polycaprolactone, *PLCL* poly(L-Lactic-co-caprolactone), *TMP* tetramethylpyrazine
